# Effectiveness of school dental screening on dental visits and untreated caries among primary schoolchildren: study protocol for a cluster randomised controlled trial

**DOI:** 10.1186/s13063-018-2619-2

**Published:** 2018-04-13

**Authors:** Haya Alayadi, Wael Sabbah, Eduardo Bernabé

**Affiliations:** 1 Division of Population and Patient Health, King’s College London Dental Institute at Guy’s, King’s College and St. Thomas’ Hospitals, London, UK; 2 Department of Community Dentistry, Faculty of Dental Hygiene Department, King Saud University, Riyadh, Saudi Arabia

**Keywords:** Mass screening, Schools, Dental caries, Randomised controlled trials, Dental care for children

## Abstract

**Background:**

Dental caries is one of the most common diseases affecting children in Saudi Arabia despite the availability of free dental services. School-based dental screening could be a potential intervention that impacts uptake of dental services, and subsequently, dental caries’ levels. The purpose of this study is to evaluate the effectiveness of two alternative approaches for school-based dental screening in promoting dental attendance and reducing untreated dental caries among primary schoolchildren.

**Methods:**

This is a cluster randomised controlled trial comparing referral of screened-positive children to a specific treatment facility (King Saud University Dental College) against conventional referral (information letter advising parents to take their child to a dentist). A thousand and ten children in 16 schools in Riyadh, Saudi Arabia, will be recruited for the trial. Schools (clusters) will be randomly selected and allocated to either group. Clinical assessment for dental caries will be conducted at baseline and after 12 months by dentists using the World Health Organisation (WHO) criteria. Data on sociodemographic, behavioural factors and children’s dental visits will be collected through structured questionnaires at baseline and follow-up. The primary outcome is the change in number of teeth with untreated dental caries 12 months after referral. Secondary outcomes are the changes in the proportions of children having untreated caries and of those who visited the dentist over the trial period.

**Discussion:**

This project should provide high level of evidence on the clinical benefits of school dental screening. The findings should potentially inform policies related to the continuation/implementation of school-based dental screening in Saudi Arabia.

**Trial registration:**

ClinicalTrials.gov, ID: NCT03345680. Registered on 17 November 2017.

## Background

Dental caries is one of the most common diseases affecting children in Saudi Arabia despite the availability of free dental services. The combination of the large burden of untreated caries among schoolchildren, low uptake of dental services when asymptomatic and the availability of free dental services makes Saudi Arabia a unique setting for evaluating school-based dental screening. Despite the availability of free dental services provided through the Ministry of Health, universities’ hospitals and health services of the Ministry of Defence, most Saudis visit the dentist only when in pain [1, 2]. Given the cultural and social norms related to symptomatic dental visits and the availability of free dental services, screening and referral of schoolchildren could be an effective intervention to promote asymptomatic dental visits and to tackle the burden of dental caries [3]. It is also an opportunity for targeting a large portion of the population with a high level of disease as a quarter of the Saudi population is younger than 15 years [4, 5]. School-based dental screening allows early contact of children with the dentist, and may subsequently, promote better compliance of children and their parents [6], which might lead to motivating asymptomatic visits, early detection and timely intervention [2].

Two recent systematic review concluded uncertainty of either harm or benefit of school-based screening in improving children’s oral health or dental attendance [7, 8]. Most of the seven randomised controlled trials (RCTs) identified among the two reviews were of high or uncertain risk of bias, especially in relation to randomisation (no allocation concealment), blinding (lack of blinded outcome assessment) and attrition (incomplete outcome data) [9–14]. The only high-quality study was conducted in the United Kingdom (UK) [15]. This was also the only RCT that examined clinical outcomes (untreated dental caries) rather than just dental attendance. The fact that around 50% of children with untreated caries referred to a dentist received treatment for the referred condition [15] suggests that dental attendance does not provide sufficient information to assess the clinical benefits of school-based dental screening. Another salient methodological issue is that most RCTs looked at short-term effects (3 to 8 months) [7, 8]. A longer follow-up period might be needed to detect clinical changes.

All seven RCTs had a limited scope to dental screening, merely devoted to providing information to parents [7, 8]. No further attempts for follow-up communication and/or provision of assistance to parents with booking appointments was included [9–15]. A successful screening programme requires more than just the screening test; it should have a supporting system to ensure that those needing treatment are able to obtain it [6, 16, 17]. The provision of free dental services does not solve the problem as shown in the UK studies [9–11, 13, 15]. These extra efforts and costs to facilitate contact of screened-positive individuals with health services must be balanced against potential gains (clinical benefits) of the screening programme. Given the above reasons, it was not surprising that systematic reviews concluded that there is a need to conduct a well-designed trial with an intensive follow-up group [7, 8]. The present trial addresses these gaps in knowledge and methodological challenges.

The primary aim is to evaluate the effectiveness of two alternative approaches for school-based dental screening (referral to a specific treatment facility versus current practice —i.e. an information letter advising parents to take their child to a dentist—) in promoting dental attendance and reducing untreated caries among primary schoolchildren in Saudi Arabia. A secondary aim is to determine the effectiveness of school-based dental screening among population subgroups (two subgroups will be specifically considered, namely children who were screened positive and children who reported being regular attenders at baseline).

## Methods

This manuscript adheres to the Standard Protocol Items: Recommendations for Interventional Trials (SPIRIT) 2013 Statement [18] for reporting of protocols for clinical trials and the Consolidated Standards of Reporting Trials (CONSORT) 2010 Statement – extension for cluster randomised trials [19].

### Trial design

This will be a cluster randomised, assessor-blinded, superiority trial with two groups running in parallel and an allocation ratio of 1:1. The trial will be conducted over 12 months, from baseline to follow-up outcome assessments. Randomisation at cluster level (school) will prevent contamination that could arise from delivering both interventions in the same school (i.e. randomisation at child level) while also accounting for variations between schools and areas in which the schools are located.

The trial is sponsored by King’s College London. It is a PhD project carried out by the principal investigator (HA) and supervised by WS and EB. The progress of the study will be closely monitored by a PhD Committee (acting as a Trial Steering Committee), including the principal investigator, the two supervisors and two independent external examiners.

### Setting and participants

The trial will be conducted across 16 primary schools in Riyadh city, Saudi Arabia. A list of all governmental primary schools in Riyadh city, Saudi Arabia, will be obtained from the Ministry of Education and stratified according to area (schools located in high-socioeconomic and low-socioeconomic areas). Schools with in-house dental clinics and participating in any dental screening/preventive programme will be excluded from the trial.

Children in selected primary schools will be considered eligible to join the trial if they meet all inclusion criteria and none of the exclusion criteria.

#### Inclusion criteria

- Children aged 6 to 11 years (1st to 5th grade at school) at baseline
- Both Saudis and non-Saudis
- Children for whom the person with parental responsibility has signed the consent form

#### Exclusion criteria

- Children in 6th grade (12-year-olds) as they would have left schools by the time of the follow-up assessment (12 months later)
- Children with any medically compromised condition such as congenital heart disease, haematological conditions, immune deficiency disease and end-stage renal disease
- Children who refuse to participate (assent) in the study

### Trial interventions

Children in both groups will be examined for dental caries. The difference between the two trial groups lies in the referral mechanism. A comparison against no intervention was disregarded due to ethical concerns (children would be examined and not informed of their oral problems). Those having untreated dental caries according to the World Health Organisation (WHO) [20] criteria will be considered screened positive and referred according to their allocated group. All others will be considered screened-negative and will not receive a referral.

After dental examinations, screened-positive children in the intervention group will be referred to a specific hospital for treatment (referral to a specific treatment facility), King Saud University Dental College (KSU-DC). Referral to this specific institution is one of the innovations in the proposed research. The triage unit at KSU-DC will provide any urgent care needed and make an appointment for subsequent care. All treatments will be provided free of charge. Screened-positive children in the comparison group will receive the currently accepted practice for school-based dental screening; that is, a letter will be sent to parents informing them of the child’s dental problem and advising to take them to a dentist (conventional screening). For both groups, referral letters will be sent to parents through children’s school bags.

### Trial outcome

Combining clinical and self-reported outcomes will provide more robust evidence on the effectiveness of school-based dental screening. While self-reported dental visits are a direct indicator of the impact of the intervention, they are a surrogate measure of clinical status and subject to measurement bias. We have, therefore, chosen a clinical measure as primary outcome as it will confirm that a dental visit occurred during the trial period. A clinical measure also allows a blinded outcome assessment (caries examination), something that could not be achieved with self-reported dental visits as participants will know which group they are in.

#### Primary outcome

The study primary outcome is the change in the number of teeth with untreated dental caries in both primary and permanent teeth over 12 months. The underlying assumption of the selected primary outcome is that students in the intervention group will visit a dentist during the trial period and as a result will have fewer untreated decayed teeth than those in the comparison group. While the use of a clinical outcome might be perceived more sensitive to services provided after referral, its use is deemed appropriate for this trial as the students will be referred to a specific institution with a system in place to handle those referred.

#### Secondary outcomes

The change in the proportion of children having teeth with untreated caries and the change in the proportion of children who reported visiting a dentist (as reported by parents and children) during the duration of the trial will be the secondary outcomes.

### Trial procedures

**Fig. 1:**
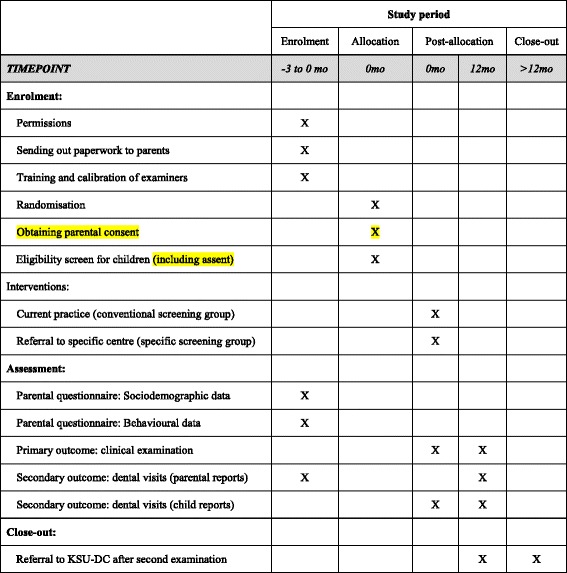
Study timeline detailing enrolment, interventions and assessments

The time schedule of enrolment, interventions, assessments is shown in Fig. 1.

#### Randomisation

Simple randomisation will be used to allocate schools to either of the two interventions within each stratum. A third-party (statistician) will generate the allocation sequence using computer-generated random numbers and will inform the principal investigator (not directly involved in data collection), who will subsequently send separate fieldwork teams to schools.

#### Blinding

Participants cannot be blinded in this study given the nature of the interventions. Children and their parents will know they are taking part in a trial (after reading the information sheet) and whether their children will be allocated to the intervention or comparison group from the referral letter. Outcome assessors will be blinded by sending separate fieldwork teams (who will not be aware of the study aims) for the intervention and comparison groups. We have also chosen untreated dental caries as our primary outcome (rather than self-reported dental visits) as the former is considered more reliable and less prone to measurement bias. Self-reported dental visits could be influenced by participants’ and their parents’ knowledge of which trial group they are in.

### Data collection

The study will obtain approval from the Saudi Ministry of Health to ensure no screening programme is implemented in the selected schools and from the Ministry of Education to both obtain the list of schools in Riyadh city and access to the selected schools. Approval will also be obtained from KSU-DC to refer children to the dental clinic.

After obtaining these approvals, the primary researcher will send invitation letters to selected schools. Refusals and non-respondent schools will be replaced with new schools randomly chosen from the same list. For schools that agree to participate, the primary researcher will organise dates and times for data collection in meetings with heads of schools.

In the schools that agree to participate, the participants’ information sheet, a written informed consent form and a structured questionnaire will be sent to parents in the children’s school bag. A text message from the school administration will be sent on the same day informing parents about the documents and encouraging participation. A new text message will be sent after 1 week to all parents thanking those who have already agreed to participate and reminding parents who have not sent the forms back to do so before the fieldwork team visits the school. The parental questionnaire has been modified from the WHO questionnaire for assessing oral health status [20] to collect data on sociodemographic characteristics (children’s age and gender, household size, parental education and family income), dental visits, oral hygiene behaviour and diet of the child. Parents will return the signed consent forms and completed questionnaires with their children. A shorter version of the questionnaire (focussing on the child’s last dental visit during the last 12 months) will be sent to all parents a week prior to the second visit to schools for the follow-up clinical examinations.

Only children with signed parental consent will be clinically examined. The fieldwork team will explain the activities to be carried out in the classroom. Signed consent forms and parental questionnaires will be collected at this stage and children’s eligibility checked. Before examining every child, the recorder will ask children whether they had visited the dentist in the last 12 months, and if so, the reason for that visit will be recorded. The same question will be asked to children during follow-up clinical assessments (‘since we last saw you’).

There will be 12 dentists doing the fieldwork under the supervision of the principal investigator. Each fieldwork team will consist of an examiner who will perform clinical examinations and a recorder who will register codes in examination forms. Individuals will switch roles to minimise visual fatigue. They have been trained and calibrated before the beginning of the trial. Mean Kappa values for inter- and intra-examiner reliability in caries diagnosis at tooth level were 0.78 and 0.86, respectively. The principal investigator will hand over referral letters to screened-positive children in girls-only schools (instructing them to put them in the school bags and taking them to their parents). A male organiser, who will not be involved in dental examinations, will do the same in boys-only schools.

Clinical examinations for dental caries will be performed according to WHO criteria that defines dental caries at the caries into dentine threshold [20]. No radiographs will be taken. Examinations will be conducted similarly at baseline and follow-up, with children sitting on a school chair, using headlamps, plane mouth mirrors and a periodontal probe. Teeth will not be brushed or professionally cleaned before examination but cotton wool rolls/buds and the periodontal probe will be used, respectively, to dry the surfaces and remove moisture/debris to facilitate visual inspection. Standard infection control measures will be in place.

After clinical examinations at baseline and follow-up, 10% of participants will be re-examined by the same fieldwork team to assess intra-examiner reliability. After clinical examinations in the follow-up assessment (at 12 months) screened-positive children in both trial groups will be referred to KSU-DC (close-out procedure).

Given the training and calibration of examiners in caries assessment, the probability of having false-positive cases is minimal. Upon referral, a standard dental check-up is always conducted as part of the treatment plan where any false-positive cases will be identified. If that occurs, the dentist will inform parents that upon further examination no treatment is required.

#### Ensuring participant retention

Given that this is a 12-month trial, baseline and follow-up data will be collected in separate school years (summer holidays in-between). This implies that there might be some losses to follow-up as children might change residence/school. We have increased the trial size accordingly to compensate for 11% attrition (i.e. derived from the proportion of children continuing in the same school the following year). Fieldwork teams will stay in schools for an entire week (as they progress screening children from one classroom to another) to give an option to those children who were absent the day that their classroom was screened. That aside, we expect a high completion rate as parental consent will be sought for both waves of data collection simultaneously. Using a clinical outcome measure will prevent further losses to follow-up as schools are captive populations. Attrition could be higher for parental questionnaires (used to estimate child dental visits). However, we are also collecting information on dental visits from the children’s perspective. Furthermore, dental visits are the secondary outcome and will not affect the main conclusion of the trial.

#### Data storage and management

The principal investigator will be responsible for all data entry and management. Personal identifiable information will be collected to regain contact with participants for the follow-up assessment and link baseline with follow-up data. This will be handled through pseudo-anonymisation, whereby the principal investigator will keep a separate file containing personal identifiable information for all participants and assign an artificial identifier (code) to each as they enter the study. Everybody else involved in the study will work with coded data when collecting or analysing information.

Data entry will be done using codes and no personal information will be kept electronically. Coded data will be entered to a password-protected device (laptop) owned by the primary researcher on an ongoing basis as data collection progresses. Once data collection has been completed, all paper forms (consents, questionnaires and clinical examination) will be transferred to a locked cabinet and electronic files transferred to a password-protected desktop computer both located at King’s College London Dental Institute. No data will be accessed by anyone other than the three members of the research team.

### Data analysis

#### Justification of sample size

Based on a previous relevant study [15], a sample size of 910 children (455 in each trial group) is required to detect a 0.50-unit difference in the number of teeth with untreated caries between the conventional screening group and specific screening group, assuming 80% statistical power, 5% significance level and intra-class correlation coefficient (ICC) for dental caries at school level of 0.03. The total sample size will be rounded up to 1010 (505 in each group) to allow for a dropout rate of up to 11%. After compensating for the clustering of children within schools, there will be six clusters per group, each cluster will include at least 75 participants.

#### Between-group comparisons

A comparison of demographic (sex, age), socioeconomic (family income, parental education) and behavioural characteristics (tooth-brushing frequency, dental attendance pattern, last dental visit, reason for last dental visit, use of fluoridated toothpaste and sugars intake) and caries levels in deciduous and permanent teeth between both intervention groups will be carried out using the chi-square test for categorical variables and a *t* test for continuous variables.

We will report absolute and relative measures of effect; that is, absolute and relative difference in means for continuous outcomes (change in number of teeth with untreated caries) and risk differences and relative risks for dichotomous outcomes (changes in the proportions of children having teeth with untreated caries and visiting the dentist). For the primary outcome (change in number of teeth with untreated caries), negative binomial regression will be used to estimate relative differences (incidence rate ratios) and predictive margins to estimate absolute differences between intervention groups. This analytical technique will allow accounting for stratification of the sample, clustering of children within schools and any baseline socioeconomic and behavioural differences between trial groups. For secondary outcomes, log-binomial regression will be used to estimate relative risks for the change in the proportions of children with untreated dental caries and who visited the dentist. Predictive margins will also be used to estimate absolute differences in each secondary outcome. Intention-to-treat analysis will be used to handle missing values in categorical outcomes. Multiple imputation will be considered as an option for continuous outcomes.

#### Supplemental analysis

Subgroup analysis will be conducted to explore the effectiveness of school-based dental screening on primary and secondary outcomes among specific subgroups. To that end, we will compare outcomes between the two trial groups among children who were screened positive, and then, among those who reported being regular attenders at baseline.

Comparisons will also be reported stratified by area (high and low socioeconomic status) and dentition (number of teeth with untreated caries in primary or permanent teeth).

## Discussion

Given the high level of children’s dental caries in Saudi Arabia, the cultural norms of only visiting the dentist when in pain [2], and the availability of free dental services through the Ministry of Health, school-based screening could be an appropriate intervention for the epidemic of childhood dental caries in Saudi Arabia. While studies in other countries have examined the impact of school screening on dental visits and dental caries and showed variation in their results [9–15], this study goes one step further by referring the children to a specific healthcare facility with a system in place to handle referred children. Compared to studies in western countries that showed no effectiveness of dental screening [9–11, 13, 15], the population of Saudi Arabia could be different as asymptomatic dental visits are uncommon [2].

The findings of the proposed research will provide important information on the potential impact of school-based dental screening on untreated dental caries and the uptake of dental services. If the research shows a positive impact it will set the scene for recommending the implementation of school-based dental screening nationally. A positive finding will also provide the grounds for continuation of any existing screening programme in other parts of the country. The findings of this study will also highlight the importance of the ongoing monitoring of such programmes and continuous evaluation of their effectiveness. Contrarily, if the study does not show any benefit, it will highlight the importance of adequately examining the effectiveness and cost-effectiveness of any existing programmes.

Using two different outcome measures, reflecting uptake of services and clinical outcome, will help us disentangle where the screening programme might have failed. An increase in use of dental services without differences in clinical outcome would reflect issues with services but not with the screening tool. On the other hand, lack of effectiveness in both uptake of services and clinical outcome will reflect problems with the overall screening process.

The selected study design addresses some of the methodological limitations of previous studies [7, 8]. Data collection on participants’ socioeconomic and behavioural factors will help accounting for baseline differences between the two trial groups (if they are unbalanced after randomisation) that might impact use of dental services [21]. However, this study must be conducted with relatively limited resources as part of PhD project and will probably not answer all relevant questions on the effectiveness of school dental screening in Saudi Arabia. Future research should build upon what we are starting in this study, by looking into cost-effectiveness analysis and reasons behind any lack of effectiveness. A national study could also provide more information on effectiveness in different parts of the country.

Finally, it is worth noting that the education system in Saudi Arabia is segregated by children’s gender. Due to cultural restrictions, female dentists cannot access boys-only schools and vice versa. Although we have recruited and trained separate teams for accessing boys- and girls-only schools, access to both types of schools are heavily dependent on approval from the Ministry of Health as the principal investigator, who will oversee the entire fieldwork, is female. This might result in a male-to-female ratio different to 1-to-1.

## Trial status

Protocol version 3 (13 March 2018). Recruitment began on 20 November 2017 and it is expected to be completed by April 2018.

## Abbreviations

ICC: Intra-class correlation coefficient
KSU-DC: King Saud University Dental College
RCT: Randomised controlled trial
UK: United Kingdom
WHO: World Health Organisation
